# Mid-Year Populations by Sex, Age, and Race and Ethnicity of U.S. Active Component Service Members, 2023–2025

**Published:** 2026-04-01

This Surveillance Snapshot describes the mid-year population for active component service members (ACSMs) of the U.S. Army, Navy, Air Force, Marine Corps, Space Force, and Coast Guard from 2023 to 2025, stratified by age, sex, and race and ethnicity. Population counts were obtained from June of each calendar year using personnel data from the Defense Manpower Data Center (DMDC) maintained in the Defense Medical Surveillance System (DMSS). Counts and percentages were stratified by sex, age group, and race and ethnicity.

In DMSS, race and ethnicity are categorized as non-Hispanic White, non-Hispanic Black, Hispanic, Asian/Pacific Islander, and American Indian/Alaskan Native, and Other. Both sex and race and ethnicity are self-reported by individual service members. As of 2021, the values for Asian/Pacific Islander and American Indian/Alaskan Native are not populated in DMSS for the Air Force and Coast Guard, and these 2 groups have been included in the ‘other’ category for this report.

The minimum and maximum mid-year populations of ACSMs ranged from 1,063,862 to 1,085,521 among men and 230,260 to 240,065 among women during the surveillance period. Demographic shifts among ACSMs varied by age and sex. In 2025, 81.8% of the active component was comprised of men, and 18.2% was comprised of women **(Table)** . Stratified by age, the proportion of women was highest among the younger than age 20 years group (21.3%) and lowest in the ages 50-54 years group (15.1%).

**TABLE.** Number of Active Component Service Members in June ^a^ , 2023–2025, Stratified by Sex, Age Group, and Race and Ethnicity

		2023				2024				2025			
		Men		Women		Men		Women		Men		Women	
Age	Race and Ethnicity	No.	%	No.	%	No.	%	No.	%	No.	%	No.	%
<20	White, non-Hispanic	32,612	51.2	5,327	35.1	30,711	47.7	5,083	31.0	32,694	46.7	5,661	30.2
	Black, non-Hispanic	9,220	14.5	3,596	23.7	9,791	15.2	4,181	25.5	10,540	15.0	4,738	25.2
	Hispanic	16,459	25.8	4,788	31.5	18,262	28.4	5,556	33.9	20,652	29.5	6,636	35.4
	Other ^b^	5,441	8.5	1,468	9.7	5,630	8.7	1,575	9.6	6,160	8.8	1,735	9.2
20–24	White, non-Hispanic	179,502	54.0	28,964	39.5	167,393	52.2	26,434	37.3	162,019	50.2	25,712	34.8
	Black, non-Hispanic	46,620	14.0	16,236	22.2	46,908	14.6	16,452	23.2	49,404	15.3	18,275	24.7
	Hispanic	73,337	22.1	19,416	26.5	74,739	23.3	19,677	27.8	79,458	24.6	21,358	28.9
	Other ^b^	32,792	9.9	8,681	11.8	31,644	9.9	8,296	11.7	31,873	9.9	8,540	11.6
25–29	White, non-Hispanic	137,542	54.8	23,472	41.0	133,008	53.9	22,504	39.9	132,841	52.6	22,471	38.7
	Black, non-Hispanic	36,939	14.7	13,058	22.8	36,711	14.9	12,994	23.0	38,749	15.3	13,694	23.6
	Hispanic	45,686	18.2	12,043	21.0	47,039	19.1	12,457	22.1	50,423	20.0	13,370	23.0
	Other ^b^	31,038	12.4	8,660	15.1	30,137	12.2	8,480	15.0	30,463	12.1	8,497	14.6
30–34	White, non-Hispanic	98,950	55.3	16,028	40.9	95,696	54.1	15,691	40.1	93,453	53.0	15,341	38.9
	Black, non-Hispanic	26,295	14.7	9,330	23.8	26,791	15.1	9,403	24.0	27,536	15.6	9,657	24.5
	Hispanic	29,064	16.2	7,135	18.2	29,635	16.8	7,364	18.8	30,772	17.5	7,760	19.7
	Other ^b^	24,675	13.8	6,672	17.0	24,733	14.0	6,671	17.0	24,479	13.9	6,724	17.0
35–39	White, non-Hispanic	83,509	58.7	11,134	41.8	81,035	57.7	11,187	41.5	79,512	56.3	11,480	40.6
	Black, non-Hispanic	19,498	13.7	6,564	24.6	19,671	14.0	6,564	24.3	20,436	14.5	7,005	24.8
	Hispanic	20,309	14.3	4,341	16.3	20,590	14.7	4,519	16.7	21,557	15.3	4,788	17.0
	Other ^b^	19,021	13.4	4,594	17.2	19,221	13.7	4,718	17.5	19,689	13.9	4,973	17.6
40–44	White, non-Hispanic	44,675	59.3	5,757	43.2	44,067	58.9	5,866	43.1	44,868	58.4	6,091	42.1
	Black, non-Hispanic	10,183	13.5	3,360	25.2	10,157	13.6	3,335	24.5	10,714	13.9	3,559	24.6
	Hispanic	10,200	13.5	1,926	14.4	10,174	13.6	2,015	14.8	10,458	13.6	2,193	15.2
	Other ^b^	10,251	13.6	2,294	17.2	10,444	14.0	2,404	17.7	10,821	14.1	2,620	18.1
45–49	White, non-Hispanic	17,168	60.6	2,013	44.9	16,265	59.9	2,052	44.7	16,171	59.4	2,234	45.6
	Black, non-Hispanic	3,746	13.2	1,114	24.9	3,542	13.0	1,128	24.6	3,556	13.1	1,152	23.5
	Hispanic	3,620	12.8	556	12.4	3,540	13.0	595	13.0	3,611	13.3	626	12.8
	Other ^b^	3,802	13.4	797	17.8	3,801	14.0	812	17.7	3,892	14.3	886	18.1
50–54	White, non-Hispanic	6,716	64.2	810	47.2	6,058	62.5	765	46.7	5,724	61.2	771	46.5
	Black, non-Hispanic	1,365	13.0	447	26.0	1,296	13.4	393	24.0	1,257	13.4	404	24.4
	Hispanic	1,026	9.8	175	10.2	1,014	10.5	178	10.9	1,040	11.1	182	11.0
	Other ^b^	1,358	13.0	284	16.6	1,320	13.6	302	18.4	1,339	14.3	302	18.2
55+	White, non-Hispanic	1,932	66.6	319	51.4	1,845	65.0	302	49.6	1,935	63.6	321	51.0
	Black, non-Hispanic	363	12.5	169	27.2	355	12.5	152	25.0	402	13.2	144	22.9
	Hispanic	212	7.3	44	7.1	228	8.0	54	8.9	268	8.8	58	9.2
	Other ^b^	395	13.6	89	14.3	411	14.5	101	16.6	439	14.4	107	17.0
Total	White, non-Hispanic	602,606	55.5	93,824	40.5	576,078	54.1	89,884	39.0	569,217	52.7	90,082	37.5
	Black, non-Hispanic	154,229	14.2	53,874	23.3	155,222	14.6	54,602	23.7	162,594	15.1	58,628	24.4
	Hispanic	199,913	18.4	50,424	21.8	205,221	19.3	52,415	22.8	218,239	20.2	56,971	23.7
	Other ^b^	128,773	11.9	33,539	14.5	127,341	12.0	33,359	14.5	129,155	12.0	34,384	14.3

Abbreviation: No. number.

a Army, Navy, Air Force, Marine Corps, Space Force, and Coast Guard.

b Includes those of American Indian / Alaska Native, Asian / Pacific Islander, and unknown racial and ethnic group.

Overall, while non-Hispanic White service members represented the largest proportion by race and ethnicity in 2025, that proportion declined over the surveillance period. The proportion of Hispanic service members increased throughout the surveillance period, continuing a recent trend. ^1^ The greatest demographic shifts are seen in the younger than age 20 years group, with the greatest percent increase in numbers among service members identifying as Black, non-Hispanic, Hispanic, and ‘other’ for both sexes. Among female service members younger than age 20 years, those identifying as Hispanic constituted the largest group (35.4%) in 2025. Similar trends are seen among male service members younger than age 20 years, where non-Hispanic White male service members represented less than half of men in that age group (46.7%), and the proportion of Hispanic men has increased since 2023 (29.5%). Among older age groups, demographic shifts are less pronounced. These data can be used to provide additional context for health surveillance analyses for U.S. ACSMs.
